# Growth indices, intestinal histomorphology, and blood profile of rabbits fed probiotics- and prebiotics-supplemented diets

**DOI:** 10.1093/tas/txab096

**Published:** 2021-06-30

**Authors:** Chinwe Uchechi Nwachukwu, Karimat Imam Aliyu, Emmanuel Olubisi Ewuola

**Affiliations:** 1 Department of Agricultural Science, School of Agriculture and Vocational Studies, Alvan Ikoku Federal College of Education, Owerri, Imo State, 460281, Nigeria; 2 Livestock Department, Ministry of Agriculture and Natural Resources, Kwara State, Nigeria; 3 Animal Physiology Laboratory, Department of Animal Science, University of Ibadan, Ibadan, Nigeria

**Keywords:** dietary treatment, growth promoter, performance, prebiotics, probiotics, rabbits

## Abstract

In a 12-week feeding trial, 32 rabbits (Chinchilla × New Zealand White; 56 days old; 691 ± 1 g body weight) were used to investigate the effect of pro- and prebiotics as growth enhancer on the growth performance, intestinal mucosal development, hematological and serum biochemical responses of rabbits. The dietary Biotronic® prebiotics and Biovet®-YC probiotics were added at 400 mg/kg and 50 mg/kg, respectively. The rabbits were housed individually and randomly assigned to four dietary treatments (*n* = 8/group; 50:50 bucks to does) including a control diet (diet 1), diet 2 (control + Biotronic® prebiotics), diet 3 (control + Biovet®-YC probiotics) and diet 4 (control + symbiotics [Biotronic® prebiotics and Biovet®-YC probiotics]). Body weight (BW), average daily gain (ADG), dry matter intake (DMI), and feed conversion ratio (FCR) were monitored. Five rabbits per treatment were used for organ assessment and intestinal histomorphology after feeding trial. Blood samples were collected for hematological and serum biochemical analysis. Results showed that supplementation of Biotronic® prebiotics and symbiotics in rabbit diet significantly (*P* < 0.05) increased final BW and ADG compared to Biovet®-YC probiotic and control diets. Kidney, lung, esophagus, gastro-intestinal tract, small and large intestines were significantly (*P* < 0.05) influenced by dietary treatments. Ileal mucosal assessment revealed that villus height (VH), villus width, villus density, crypt depth (CD), and VH:CD ratio of rabbits fed Biotronic® prebiotic and symbiotic diets were similar and significantly (*P* < 0.05) higher than those rabbits fed control and Biovet®-YC probiotic diets. Packed cell volume of rabbits fed symbiotic and control diets was significantly (*P* < 0.05) higher than those fed Biotronic® prebiotic and Biovet®-YC probiotic diets. This study suggests that Biotronic® prebiotics and its combination with Biovet®-YC probiotics are good alternative growth promoting feed additives in rabbit nutrition. They improved performance, intestinal development and blood profiles and aid feed digestion, nutrient absorption and utilization in rabbits.

## INTRODUCTION

Increase in the human population and inadequate animal products are major challenges to meet the demand of animal protein from livestock production. Rabbit farming provides numerous advantages over large animals since they are proficient in reproduction, higher in protein content, lower in fat/cholesterol levels and generate a steady source of income compared with many other farm animals (Appiah et al., 2011). Biotechnological treatment to enhance the nutritive value of rabbit feed has improved the digestibility of fibrous agricultural by-products either by direct use of microorganisms or microbial enzymes. Besides, inclusion of live yeast in animal feeds have shown to improve digestibility, feed efficiency, growth rate, animal performance, egg/meat production, animal health, reduce pathogenic bacteria and negative environmental impact when used in farm animals (Ezema and Eze, 2012). Other feed additives with similar potentials are on trial, since enhancing growth is critical to farmers to boost their production so as to meet protein demand of the populace and increase profit margin as return on investment within a short possible time. In addition, there is also interest in non-antibiotic growth promoters that might have similar positive effects in controlling pathogenic organisms in the gut of food producing animals in order to increase their production performance (Sengupta and Chattopadhyay, 2012; Laxminarayan et al., 2013).

Among many promising growth promoting agents are plant extracts from herbs and spices, organic acids, probiotics, prebiotics, symbiotics, and enzymes as feed additives that have been hypothesized to be effective as natural growth promoters in animal diets. These feed additives are promising natural growth promoters that should be investigated in rabbits for their effects on animal performance, health, immunity, and welfare (Ewuola et al., 2011, 2012). The prebiotics and probiotics act to activate beneficial microbes in the gut, improve the immune system, reduce the gut pH, induce protective gut mucus, demonstrate positive antimicrobial properties, and improve the intestinal histomorphometry, feed digestibility, and nutrient absorption in some farm animals (Hamasalim, 2016; Likotrafiti et al., 2016). Effective gastrointestinal functionality and health is a complex system including diet, effective structure and function of the gastrointestinal barrier, host interaction with the gastrointestinal microbiota, effective digestion and absorption of feed, effective immune status, and neuroendocrine function of the gut (Ewuola et al., 2012; Celi et al., 2019).

The gastrointestinal tract (GIT) is the main digestive and absorptive organ in the animal. The GIT permits the uptake of dietary substances into systemic circulation and it excludes pathogenic compounds (Clissold et al., 2010). It also harbors an extremely complex microbiota which has a profound impact on animal’s health, immunological, respiratory, and gastrointestinal functions (Floch et al., 2011). The intestinal epithelium acts as a natural barrier against pathogenic bacteria and toxic substance that are present in the intestinal lumen. The absorption ability of the GIT is dependent on the mechanisms that occur in the intestinal mucosa, the manipulation of probiotics (microbial supplement comprised of specific bacteria or fungi together) with prebiotics (non-digestible ingredients that are beneficial to the host because to the host they selectively stimulate growth and/or the activity of certain bacteria in the intestine) have been used to improve performance and intestinal efficiency in some animals (Hofacre et al., 2003; Pelicano et al., 2005).

Most microorganisms used in probiotics are strains of beneficial gram-positive bacteria of the genera *Bacillus, Enterococcus, Lactobacillus, Pedococcus, Bifidobacterium*, and *Streptococcus*. Some yeast and fungi are used most times like strain of *Saccharomyces cerevisiae (*Marco et al., 2006) while the prebiotics are fructo-, galacto-, and trans-galacto-oligosaccharides which are promising additives (Scott et al., 2013; Bindels et al., 2015). These dietary feed additives could have measurable effects on animal health, performance, and immunity as nutritional evaluation and animal status can be measured from blood and tissue constituents (Ewuola et al., 2011, 2012). Thus, this study was designed to investigate the effect of dietary Biotronic® prebiotics and Biovet®-YC probiotics on growth performance, organ weights, intestinal histomorphological development, and blood profile of rabbits.

## MATERIALS AND METHODS

### Ethical Approval, Experimental Location, and Animal Study

The institutional research and ethics committee (University of Ibadan, Ibadan, Nigeria) approved the animal study and its experimental protocols, which was performed in accordance with standard guidelines of University Animal Scientific procedures [Animal (Scientific procedures) Act 1986]. The animals were handled in accordance with the National Institutes of Health (NIH Publication No. 85-23; revised 1996) guidelines for care and use of laboratory animals. The rabbits were brought from Covenant Farm, Gbolasire area, Iwo road, Ibadan, Oyo State. The study was carried out at the rabbit unit of the Teaching and Research Farm, University of Ibadan, Ibadan, Nigeria. The site is located at latitude 7°20′N and 3°50′E, and 200 m above sea level. The basal diet was made isocaloric on a dry matter as-fed basis for the formulated diets to meet the nutrient requirements of the rabbits (National Research Council, 1977). The basal feed ingredients (diet 1) and proximate composition are presented in Table 1. According to the manufacturer, each kg of Biotronic® prebiotics (Biomin) contains blended buffer of *fructo-oligosaccharides*, phytochemicals and organic acids, while Biovet®-YC probiotics (Vetoquinol) contains *Saccharomyces boulardii—*30,000 million c.f.u; *Lactobacillus acidophilus—*45,000 million c.f.u; Live yeast culture of *Saccharomyces cerevisiae sc*-47*—*3,00,000 million c.f.u; Alpha amylase*—*5 g; Sea weed extract*—*100 g; excipients*—*quantum satis (q.s.). The rationale for selecting Biotronic® prebiotics and Biovet®-YC probiotics supplement for the study was the potential and beneficial content of the products. Each of feed additives were included into the basal diet (diet 1) as diets 2, 3, and 4 containing Biotronic® prebiotics (Biomin, Austria) included at 400 mg/kg; Biovet®-YC probiotics (Vetoquinol, India) included at 50 mg/kg; and symbiotics (combination of Biotronic® at 400 mg/kg + Biovet®-YC at 50 mg/kg) respectively. The feed additives were in powdered form. The test diets were prepared by mixing the test ingredients with a small proportion of the formulated diet and later mixed with the entire diet on treatment basis as the animal experimental diets and left at room temperature overnight before the feeding trial. The proximate composition of the basal diet (Table 1) was analyzed using empirical procedure with Kjeldahl method (Association of Official Analytical Chemists, 1990); dry matter (Method 930.15), crude protein (Method 954.01), ether extract (Method 920.39), crude ash (Method 942.05) as previously published (Ewuola et al., 2011). The metabolizable energy of the prebiotic was accounted in the proximate analysis and nutrient digestibility done in this study. A total of thirty-two (32) rabbits (Crossbred: Chinchilla × New Zealand White) with average body weight of 691±1.0g (56 days old) were randomly assigned to the four dietary treatments such that each treatment had 8 animals (50:50 ratio of bucks to does per group) housed individually in 35 × 40 × 40 cm hutch in 12-week feeding trial. All animals were given 1 week of acclimatization to animal housing conditions before being used for the study. The experimental animals were maintained under a standardized pathogen-free animal house conditions of standard 12-h natural light/dark photoperiod per day at an environmental ambient temperature of 25 ± 2 °C. The animals were fed *ad libitum* twice daily at 08:00 h and 16:00 h. All the animals were observed daily throughout the feeding trials. For each animal, feed (300 g) was weighed in, and the residual feed was weighed out the following morning and recorded daily.

**Table 1.** Feed ingredients and chemical composition of basal diet for growing rabbits

Feed ingredients	Basal diet, as fed, %
Maize	30.00
Soybean meal	25.00
Wheat bran	9.00
Rice husk	30.00
Fishmeal	3.00
Dicalcium phosphate	2.00
Salt	0.50
Premix (Vit-Min)*	0.45
Methionine	0.03
Lysine	0.02
Proximate composition	
Metabolizable energy, kcal/kg	2744
Dry matter, g/kg	870.47
Crude protein, g/kg	180.16
Crude fibre, g/kg	120.64
Ether extract, g/kg	110.62
Ash, g/kg	120.05

*Note*: Ewuola et al. (2011).

*Composition of premix per kg contained Vitamin A 12,000,000 IU, Vitamin D3 2,500,000 IU, Vitamin E 10,000 mg, Vitamin K3 2500 mg, Vitamin B1 1000 mg, Vitamin B2 4000 mg, Vitamin B6 1500 mg, Vitamin B12 10 mg, Pantothenic acid 10,000 mg, Nicotinic acid 20,000 mg, Folic acid 1000 mg, Biotin 50 mg, Choline chloride 500 mg, Manganese 60 mg, Zinc 55 mg, Selenium 100 mg, Iodine 1000 mg, Iron 35 mg, Copper 10 mg, Cobalt 250 mg, Antioxidant, and Carrier limestone CaCO_3_. Premix supplied by Animal care, Nigeria.

### Experimental Design

Thirty-two rabbits of both bucks and does were randomly assigned to the four dietary treatments such that each treatment had eight animals per group in 12-week trial. Animals were randomly allocated to experimental groups based on body weight and gender. The number of animals per group were calculated based on the papers (Charan and Kantharia, 2013; Du Sert et al., 2020). Data on growth performance of previous experiment (Phuoc and Jamikorn, 2017) have revealed mean difference between control and experimental groups of 171 and standard error of the mean of 119. Sample size calculation was based on 80% confidence interval in most parameters which resulted in eight animals per group in our study. Sample size for organ weight and intestinal development analysis were limited to *n* = 5 in each group in order to keep the number of animals to a minimum based on the mean difference of 75 and standard derivation of 25 from villi length data of previous experiment (Liu et al., 2019). All animals used in the different experimental groups were assessed blindly.

### Growth Indices Measurement

All rabbits per treatment (*n* = 8/both genders) were used for growth performance evaluation. Initial and final body weight, ADG, DMI, and FCR of individual rabbits were measured and recorded. The FCR was calculated by dividing the DMI with ADG.

### Organ Assessment

A total of five rabbits per treatment were selected for the organ assessment. At the end of the 12-week feeding trial, all the rabbits were fasted for 12 h, euthanized by barbiturate overdose, and then slaughtered for further assessment after recording individual pre-slaughter live weight. The selected rabbits were stunned, sacrificed, skinned, and eviscerated for organ parameters. Gastrointestinal tract was separated and weighed. The liver, heart, lung, kidney, and pancreas were separated and weighed. Slaughtering and dissection were carried out according to World Rabbit Science Association recommendations (Blasco and Ouhayoun, 1996).

### Ileal Mucosal Development Assessment

Five rabbits per treatment were used for ileal mucosal assessment after they have been sacrificed. Tissue samples (2–3 mm-long cross section) were taken from the ileal, duodenal and jejunal tissues of the small intestine, carefully flushed with distilled water to remove the digesta, fixed in 10% formalin for 6 hours, dehydrated in a graded alcohol (70%, 90%, and 100%) series, cleared with methyl benzoate, and then paraffin-embedded according to standard procedures for histological analysis (Suvarna et al., 2018). Hematoxylin and eosin staining was performed to determine the intestinal histomorphology of the duodenum, jejunum, and ileum of the small intestine. The tissue blocks were serially sectioned at µm and mounted onto poly-l-lysine-coated glass slides. Every fifth and tenth slide sections were selected for histological analysis for hematoxylin and eosin staining. Two images from each section were taken at different view field (10 images per each animal) were captured at an objective lens magniﬁcation of 20×. All images were captured using a light microscope and camera (Leica Microsystems, Germany). The images were analyzed using Image J software and all data were recorded and processed in Microsoft Excel spreadsheets (Microsoft Office, 2013). Parameters measured include villus height, villus width, villus density, and crypt depth in ileum of the gastrointestinal tract. Fifty readings of villus height, villus width, and crypt depth were taken per treatment. Villus height was measured from the apical to the basal region, which corresponds to the superior portion of the crypts. Crypts were measured from the base until the region of transition between the crypt and the villus.

### Blood Collection and Analysis

Blood samples were collected from all the animals (*n* = 8/treatment/both genders) before slaughter for hematology and serum biochemical analyses at the end of the feeding trial. About 5 mL of blood was sampled between 08:00 and 09:00 h from the marginal ear vein of each rabbit into sterile vacutainer tubes with and without anticoagulant ethylene diamine tetra-acetic acid (EDTA) for hematological and serum biochemical analysis, respectively. The serum was decanted after centrifugation and kept in a –20 °C fridge until serum biochemical and enzyme activity analyses. Hematological indices such as erythrocyte counts, leucocyte counts, hemoglobin (Hb) concentration, packed cell volume (PCV) were determined according to a previously described protocol. Total leukocyte counts were determined using a Neubauer hemocytometer after dilution. Blood constants: Mean Corpuscular Volume (MCV), Mean Corpuscular Hemoglobin (MCH), and Mean Corpuscular Hemoglobin Concentration (MCHC) were determined using established formulae (Jain, 1986). Serum total protein was determined using the Biuret method (Kohn and Allen, 1995). Albumin was determined using the Bromocresol Green method (Wells et al., 1985). The globulin concentration was obtained by subtracting albumin from the total protein while the albumin/globulin ratio was obtained by dividing the albumin value by the calculated globulin value.

### Statistical Analyses

The effect of growth performance (*n* = 8), organ weights and intestinal mucosal development (*n* = 5), hematological and serum biochemical responses (*n* = 8) of rabbits within treatments were analyzed. All the data were checked for normality and heterogeneity of variance before statistical analysis. SAS package was used to analyze the statistical significance (*p* < 0.05) of the main effect of treatment by one-way ANOVA in a completely randomized-block design and a post-hoc analysis of Duncan’s multiple range tests was used to determine differences between treatment means (SAS Institute, 1990). The data were blocked by weeks of trial and the number of replicates within treatment. Data in the text and tables are presented as means with standard error of mean.

## RESULTS

The growth performance of rabbits fed dietary Biotronic® Prebiotics, Biovet®-YC Probiotics and symbiotic are shown in Table 2. Supplementation of Biotronic® prebiotics and symbiotics in rabbit diets increased (*P* < 0.05) the final body weight (1.810 kg and 1.824 kg, respectively) and ADG (98.14 g and 95.71 g, respectively), compared to the Biovet®-YC probiotic diet (1.708 kg and 81.33 g) and the control (1.710 kg and 80.71 g) and also improved the feed conversion ratio (Table 2). The DMI was significantly (*P* < 0.05) higher in rabbits fed the prebiotics and symbiotics increased (*P* < 0.05) compared to the probiotic and control diets.

**Table 2.** Growth indices of rabbits fed dietary prebiotics, probiotics, and symbiotics

Treatments						
Parameter	Diet 1	Diet 2	Diet 3	Diet 4	SEM	*P*-values
Initial live weight, g	691.00	692.00	690.00	690.00		
Final live weight, g	1710^*a*^	1810^*b*^	1708^*a*^	1824^*b*^	115.71	0.03
Dry matter intake, g	94.70^*a*^	96.27^*b*^	94.47^*a*^	97.37^*b*^	3.10	0.02
Average daily gain, g	80.71^*a*^	95.71^*b*^	81.33^*a*^	93.14^*b*^	0.62	0.004
Feed conversion ratio	1.73^*b*^	0.99^*a*^	1.16^*b*^	0.99^*a*^	0.17	0.001

*Note*: Ewuola et al. (2011).

^*a*,*b*^Mean in the same row with different superscripts are significantly (*p* < 0.05) different. Treatments/diets (*n* = 8/group; both genders: 50:50 ratio of bucks to does). SEM, standard error of mean.

Diet 1: Basal diet (control).

Diet 2: Diet 1 + prebiotics (Biotronic® at 400 mg/kg).

Diet 3: Diet 1 + probiotics (Biovet®-YC at 50 mg/kg).

Diet 4: Diet 1 + symbiotics (combination of Biotronic® at 400 mg/kg + Biovet®-YC at 50 mg/kg).

The organs weight of rabbits fed dietary Biotronic® Prebiotics, Biovet®-YC Probiotics and symbiotic are shown in Table 3. Various internal organ weights examined were not significant except kidney and lung which were significantly (*P* < 0.05) influenced among the dietary treatments. Kidney and lung weights of rabbits fed diet 2 and diet 3 were significantly (*P* < 0.05) higher than those fed diet 4 and control rabbits.

**Table 3.** Relative organ weights of rabbits fed dietary prebiotics and probiotics

Treatments						
Parameter*	Diet 1	Diet 2	Diet 3	Diet 4	SEM	*P*-values
Heart, %	0.22	0.24	0.26	0.27	0.02	0.89
Kidney, %	0.49^*a*^	0.56^*b*^	0.55^*b*^	0.54^*a*,*b*^	0.01	0.04
Lung, %	0.49^*a*^	0.64^*b*^	0.65^*b*^	0.52^*a*,*b*^	0.04	0.02
Liver, %	2.51	2.69	2.58	2.38	0.01	0.60
Pancreas, %	0.13	0.05	0.04	0.03	0.05	0.99

^*a*,*b*^Mean in the same row with different superscripts are significantly (*p* < 0.05) different.

Treatments/diets (*n* = 5/group; both genders; 3 males: 2 females). SEM, standard error of mean.

*The values are relative to the live weight.

Diet 1: Basal diet (control).

Diet 2: Diet 1 + prebiotics (Biotronic® at 400 mg/kg).

Diet 3: Diet 1 + probiotics (Biovet®-YC at 50 mg/kg).

Diet 4: Diet 1 + symbiotics (combination of Biotronic® at 400 mg/kg + Biovet®-YC at 50 mg/kg).

The length of gastro-intestinal tract of rabbits fed dietary Biotronic® Prebiotics, Biovet®-YC Probiotics and symbiotic are presented in Table 4. The gut morphometry length (cm) of rabbits were significantly (*P* < 0.05) different among the treatments except caecum and stomach. The esophagus, small and large intestines, gastro-intestinal tract were significantly (*P* < 0.05) influenced by dietary treatments. The esophagus of rabbits fed diets 3 and 4 were higher (*P* < 0.05) than those rabbits fed the control and prebiotic diets. The gastrointestinal tract of rabbits fed diets 2 and 4 were higher (*P* < 0.05) than those rabbits fed the control diet and diet 3. The small intestine of rabbits fed diet 4 was higher (*P* < 0.05) than those fed diets 2 and 3, but similar to the control diet. The large intestine of rabbits fed diet 4 was higher (*P* < 0.05) than those fed diets 2 and 4, but similar to the control diet.

**Table 4.** Gut morphometry length of rabbits fed dietary prebiotics and probiotics

Treatments						
Parameter	Diet 1	Diet 2	Diet 3	Diet 4	SEM	*P*-values
Cecum, cm	11.20	11.28	11.58	10.67	0.40	0.98
Stomach, cm	2.90	2.86	2.84	2.83	0.17	0.87
Esophagus, cm	2.73^*a*^	2.88^*a*,*b*^	3.27^*b*^	3.30^*b*^	0.29	0.04
Small intestine, cm	57.92^*a*^	59.68^*a*,*b*^	59.38^*a*,*b*^	61.73^*b*^	0.98	0.02
Large intestine, cm	21.42^a^	23.43^*a*,*b*^	24.40^*b*^	23.82^*a*,*b*^	0.78	0.03
Gastro-intestinal tract, cm	458.84^c^	512.22^***b***^	461.36^c^	494.88^*a*^	27.53	0.01

^*a*,*b*^Mean in the same row with different superscripts are significantly (*p* < 0.05) different.

Treatments/diets (*n* = 5/group; both genders; 3 males: 2 females). SEM, standard error of mean.

Diet 1: Basal diet (control).

Diet 2: Diet 1 + prebiotics (Biotronic® at 400 mg/kg).

Diet 3: Diet 1 + probiotics (Biovet®-YC at 50 mg/kg).

Diet 4: Diet 1 + symbiotics (combination of Biotronic® at 400 mg/kg + Biovet®-YC at 50 mg/kg).

Ileal mucosal development of rabbits fed Biotronic® Prebiotics and Biovet®-YC Probiotics based diets is presented in Table 5. The results of villus height (VH), villus width (VW), villus density (VD), and crypt depth (CD) followed the same pattern as the growth responses. The VH, VW, VD, and CD of rabbits fed diets 2 and 4 were higher (*P* < 0.05) than those rabbits fed the control diet and diet 3, while animals on the control diet and diet 3 were similar.

**Table 5.** Ileal mucosal developmental of rabbits fed dietary prebiotics and probiotics diets

Treatments						
Parameter	Diet 1	Diet 2	Diet 3	Diet 4	SEM	*P*-values
Villus height, µm	413.88^*a*^	614.14^*b*^	359.61^*a*^	620.50^*b*^	25.16	0.01
Villus width, µm	106.04^*a*^	164.60^*b*^	123.90^*a*^	142.71^*a*^	9.72	0.04
Villus density*	17.90^*a*^	24.70^*b*^	17.35^*a*^	23.95^*a*^	0.93	0.02
Crypt depth, µm	116.25^*a*^	133.73^*b*^	104.65^*a*^	139.14^*a*^	5.49	0.004
VH:CD	3.56^*b*^	4.79^*b*^	3.49^*a*^	4.46^*b*^	0.45	0.02

^*a*,*b*^Mean in the same row with different superscripts are significantly (*p* < 0.05) different.

*Villus density = Villus number/1,368,655 µm^2^, VH, villus height; CD, crypt depth.

Treatments/diets (*n* = 5/group; both genders; 3 males: 2 females). SEM, standard error of mean.

Diet 1: Basal diet (control).

Diet 2: Diet 1 + prebiotics (Biotronic® at 400 mg/kg).

Diet 3: Diet 1 + probiotics (Biovet®-YC at 50 mg/kg).

Diet 4: Diet 1 + symbiotics (combination of Biotronic® at 400 mg/kg + Biovet®-YC at 50 mg/kg).

The hematological parameters of rabbits fed dietary Biotronic® Prebiotics, Biovet®-YC Probiotics and symbiotic are shown in Table 6. There were no differences among diets for RBC, WBC, MCH, and MCHC. However, PCV and MCV of rabbits fed diet 4 (symbiotic diet) was higher (*P* < 0.05) than those fed diets 2 and 3, but similar to the control.

**Table 6.** Hematological parameters of rabbits fed dietary prebiotics, probiotics, and symbiotics

Treatments						
Parameter	Diet 1	Diet 2	Diet 3	Diet 4	SEM	*P*-values
RBC, × 10^6^/mm^3^	5.28	4.79	4.74	5.54	0.42	0.35
WBC, × 10^6^/mm^3^	3.26	2.97	3.41	3.56	0.27	0.59
HB, g/dL	16.82	14.67	15.73	16.35	1.54	0.41
PCV, mL %	42.50^*a*^	41.50^*b*^	41.67^*b*^	43.50^*c*^	0.31	0.04
MCV, µµ ^3^	820.80^*a*^	649.85^*b*^	888.74^*c*^	794.05^*b*^	46.83	0.02
MCH, µµg	3.17	3.10	3.32	2.99	0.16	0.45
MCHC, %	39.57	35.36	37.77	37.57	3.67	0.75

^*a-c*^Mean in the same row with different superscripts are significantly (*P* < 0.05) different.

RBC, red blood cell; WBC, white blood cell; PCV, packed cell volume; HB, hemoglobin; MCV, mean corpuscular volume; MCH, mean corpuscular hemoglobin; MCHC, mean corpuscular hemoglobin concentration. Treatments/diets (*n* = 8/group; both genders: 50:50 ratio of bucks to does). SEM, standard error of mean.

Diet 1: Basal diet (control).

Diet 2: Diet 1 + prebiotics (Biotronic® at 400 mg/kg).

Diet 3: Diet 1 + probiotics (Biovet®-YC at 50 mg/kg).

Diet 4: Diet 1 + symbiotics (combination of Biotronic® at 400 mg/kg + Biovet®-YC at 50 mg/kg).

The serum proteins and alkaline phosphatase of rabbits fed dietary Biotronic® Prebiotics and Biovet®-YC Probiotics are shown in Table 7. The serum proteins of the rabbits were not influenced (*P* > 0.05) by the dietary treatments except globulin and albumin/globulin ratio. The same trend was observed for alkaline phosphatase activity in rabbits fed experimental diets. Globulin was higher (*P* < 0.05) in rabbits fed the diet 3 and the basal (control) diet than those fed diets 2 and 4. The albumin/globulin ratio of rabbits fed the control diet (2.11) was higher (*P* < 0.05) than those fed diet 3. However, the values were still within the physiological range reported by CCAC (1980) for normal rabbits.

**Table 7.** Serum biochemical parameters of rabbits fed dietary prebiotics and probiotics diets

Treatments						
Parameter	Diet 1	Diet 2	Diet 3	Diet 4	SEM	*P*-values
Total protein (g/dL)	4.74	5.37	5.65	5.65	0.33	0.75
Albumin (g/dL)	3.25	2.89	3.28	3.23	0.20	0.55
Globulin (g/dL)	1.63^*a*^	1.78^*b*^	2.22^*c*^	1.95^*b*^	0.18	0.03
Albumin/globulin ratio	2.11^*c*^	1.58^*b*^	1.35^*a*^	1.82^*b*^	0.18	0.003
Alkaline phosphatase (i.u/L)	21.46	21.68	23.82	23.18	1.28	0.87

^a-c^Mean in the same row with different superscripts are significantly (*P* < 0.05) different.

Diet 1: Basal diet (control).

Diet 2: Diet 1 + prebiotics (Biotronic® at 400 mg/kg).

Diet 3: Diet 1 + probiotics (Biovet®-YC at 50 mg/kg).

Diet 4: Diet 1 + symbiotics (combination of Biotronic® at 400 mg/kg + Biovet®-YC at 50 mg/kg).

## DISCUSSION

The present feeding trial has provided evidence that the dietary inclusion of Biotronic® Prebiotics and its combination with Biovet®-YC Probiotics (symbiotics) in rabbit diets made the animal to utilized the diet better as they used lesser quantity of feed to gain unit weight compared to other treatments. Similar observation on the beneficial effects of these feed additives on weight gain and feed conversion ratio were reported by some researchers in farm animals like poultry and pigs (Abdel-Hamid and El-Tarabany, 2019; Dela Cruz et al., 2019). A significant positive effect on body weight and feed conversion ratio of broiler chickens was observed when given a prebiotic (*Mannan oligosaccharide*) plus an antibiotic growth promoter (copper sulfate) (Çınar et al., 2009). Research investigations have shown that dietary supplements (probiotic, prebiotic, organic acids, and their various combinations) improved body weight compared with the control to a similar extent other animal species which is in agreement with the results obtained in this research study (Bozkurt et al., 2009). Reports have showed that diets containing prebiotics achieved improved performance in poultry like other performance enhance feed additives, and that prebiotics and symbiotics were superior to probiotics in improving broiler chickens performance (Celi et al., 2019; Shirani et al., 2019). Findings from this study were at variance with the report that diets supplemented with probiotics, phytobiotics and symbiotics had no effect (*P* > 0.05) on body weight, weight gain, feed intake and feed conversion efficiency of broiler chickens (Jung et al., 2008; Erdoğan et al., 2010).

The significant difference in organ weights obtained in this study does not corroborates the earlier findings who reported that prebiotics and probiotics have no significant effect on carcass and organ characteristics of rabbits (Bhatt et al., 2017; Ayyat et al., 2018). However, Mohan et al. (1996) reported that prebiotic and probiotic supplementation to diets caused a significant decrease on the liver weight of male broiler chickens when compared to the control treatment (Mohan et al., 1996). There are a lot of discrepancy in the results of some pre-and pro-biotic studies that might be related to the dosage administration of probiotics and prebiotic inclusion, animal species, and study population (e.g. in age, gender, weight, or breed), strains of microorganism used and composition of diets (Midilli et al., 2008).

There was a considerable increase in the growth of the villi in the gastrointestinal tract of the rabbits on the prebiotic and symbiotic diets in this study. This may be attributed to the synergic effect of probiotics and prebiotics, and also the selective stimulating ability of prebiotics alone. The main site where feed digestion and absorption takes place in the body system has been reported to be small intestine (Awad et al., 2008). Absorption rates are driven by villi on the mucosa layer of the intestinal wall with the help of the enterocytes, enteroendocrine cells and goblet cells. In the present study, increased villus height suggests an increased surface area capable of greater absorption of available nutrients (Adhikari et al., 2018; Salah et al., 2019). This is in line with the increased villus height reported in the ileum when *Xylo-oligosaccharide* with *Saccharomyces cerevisiae* were fed to animals (Marinho et al., 2007). In addition, crypts between villi in the intestine produce enterokinase as a precursor to pepsinogen, which controls the production of pepsin that in turn digests protein in the gastrointestinal tract. The results implicate protein digestibility as a candidate for significant increase in final live weight as reported for animals on the prebiotic and symbiotic diets in this study. Marinho et al. (2007) reported that crypt width was highest in the duodenum for a control group, but no significant differences were found in the jejunum and ileum. Awad et al. (2009) also reported increased villus height and villus height:crypt depth ratio in duodenum and ileum, suggesting an increased epithelial cell turnover (Awad et al., 2009).

This corroborates the result of histological study reported by Awad et al. (2009) with the addition of symbiotics that increased the villus height/crypt depth ratio and villus height in ileum compared with the controls. It is understood that greater villus height is an indicator that the function of intestinal villi is activated (de Souza Andrade et al., 2019). The Biotronic® prebiotic and Biovet®-YC probiotic feed additives used in this investigation had no negative influence on microflora population in the intestinal tract of the rabbits. The numbers of beneficial organisms were higher in the GIT of rabbits than the pathogenic organisms (Ewuola et al., 2012). These results clearly indicate a selective positive effect of the Biotronic® Prebiotics, Biovet®-YC Probiotics and symbiotics on the beneficia GIT microflora. The result obtained from the study imply that components of the dietary Biotronic® Prebiotics and Biovet®-YC Probiotics fed to rabbits help to maintain the microflora balance of the intestinal tract of rabbits resulting in more efficient utilization of nutrients from the feed, more intensive processes of protein metabolism, better health status, and improved immune system response.

Hematological parameters appraise the health status of animals (Johnstone et al., 2017). They are both an index and direct reflection of the effect of dietary treatments on the animals in term of type and quantity of ingested material available for the animal to meet its physiological, biochemical, and metabolic requirement (Lucas, 1998). The result of hematological indices from this study was an indication that the health status of the animals was normal, with no adverse effects from any pathological organisms in the gut. High packed cell volume in animals on the symbiotics diet showed normal red blood cell counts and hemoglobin concentration indicating that the animals are not anemic. Similar evidence has been reported in other farm animal species like sheep, rats (Salahuddin et al., 2013), pigs (Czech et al., 2010), and chicken (Al-Saad et al., 2014). Aboderin and Oyetayo, 2006 also reported general increase in packed cell volume of rats dosed with *Lactobacillus plantarum* (Aboderin and Oyetayo, 2006).

Serum biochemical analysis is used to determine the severity of heart attack, liver damage and to evaluate protein quality and amino acid utilization in animals (Cui et al., 2019). The total protein and albumin values among the diets were within the normal physiological range for rabbits which was an indication of adequate nutrient utilization by the experimental animals. At variance with this result was the significantly increase in the serum protein of chicken fed prebiotics additives for 39 days as previously reported (Sugiharto et al., 2018). Mateova et al. (2009) observed a decrease in the activity of alkaline phosphatase and aspartate aminotransferase which can indicate improved metabolism of osteogenous mineral substances (Mátéová et al., 2009). Another study recorded that the blood protein of the treated groups of chickens with probiotics were not significantly different from the control (Alkhalf et al., 2010).

The limitation of the study was that graded level of each test ingredient was not included and evaluated to determine optimum inclusion level for rabbits. Furthermore, the study was limited as the test products had multiple microorganisms and non-digestible ingredients contained inside each content that might have acted on multiple functions that were not evaluated in the study. Therefore, further experiment could be conducted to look at each specific specie of microorganism and non-digestible contained in the products on rabbits.

## SUMMARY

The result obtained in this study showed that the Biotronic® prebiotic and Biovet®-YC probiotic growth promoting agents did not disrupt the activities of the tissues, organs and blood and were safe to be used in rabbits’ nutrition. The diets did not show any feed toxicity but instead demonstrated beneficial effect in animal feed consumption, absorption and utilization. The study has specifically demonstrated that Biotronic® prebiotic and Biovet®-YC probiotic used as a growth promoting feed additives in this study possessed the potentials of maintaining good health status in animals and improved growth performance, organ weights, intestinal histomorphology, hematological and serum proteins in rabbits.
